# How Art Changes Your Brain: Differential Effects of Visual Art Production and Cognitive Art Evaluation on Functional Brain Connectivity

**DOI:** 10.1371/journal.pone.0101035

**Published:** 2014-07-01

**Authors:** Anne Bolwerk, Jessica Mack-Andrick, Frieder R. Lang, Arnd Dörfler, Christian Maihöfner

**Affiliations:** 1 Department of Neurology, University Hospital Erlangen, Erlangen, Germany; 2 Department of Physiology and Pathophysiology, Friedrich-Alexander-University Erlangen-Nürnberg, Erlangen, Germany; 3 Education Department of the Museums in Nuremberg, Nuremberg, Germany; 4 Institute of Psychogerontology, Friedrich-Alexander-University Erlangen-Nürnberg, Nuremberg, Germany; 5 Department of Neuroradiology, University Hospital Erlangen, Erlangen, Germany; Beijing Normal University, Beijing, China

## Abstract

Visual art represents a powerful resource for mental and physical well-being. However, little is known about the underlying effects at a neural level. A critical question is whether visual art production and cognitive art evaluation may have different effects on the functional interplay of the brain's default mode network (DMN). We used fMRI to investigate the DMN of a non-clinical sample of 28 post-retirement adults (63.71 years ±3.52 SD) before (T0) and after (T1) weekly participation in two different 10-week-long art interventions. Participants were randomly assigned to groups stratified by gender and age. In the visual art production group 14 participants actively produced art in an art class. In the cognitive art evaluation group 14 participants cognitively evaluated artwork at a museum. The DMN of both groups was identified by using a seed voxel correlation analysis (SCA) in the posterior cingulated cortex (PCC/preCUN). An analysis of covariance (ANCOVA) was employed to relate fMRI data to psychological resilience which was measured with the brief German counterpart of the Resilience Scale (RS-11). We observed that the visual art production group showed greater spatial improvement in functional connectivity of PCC/preCUN to the frontal and parietal cortices from T0 to T1 than the cognitive art evaluation group. Moreover, the functional connectivity in the visual art production group was related to psychological resilience (i.e., stress resistance) at T1. Our findings are the first to demonstrate the neural effects of visual art production on psychological resilience in adulthood.

## Introduction

Recent research on visual art has focused on its psychological and physiological effects, mostly in clinical populations. It has shown that visual art interventions have stabilizing effects on the individual by reducing distress, increasing self-reflection and self-awareness, altering behaviour and thinking patterns, and also by normalizing heart rate, blood pressure, or even cortisol levels [1], [2], [3], [4], [5]. The extent to which visual art may also affect the functional neuroanatomy of the healthy human brain remains an open question.

A few fMRI studies have addressed the neural correlates of novel visual form production or have focused on the aesthetic experiences of visual artwork, the activation of the reward circuit by visual art perception for example [6]. Distinct brain areas of a certain resting state network, the default mode network (DMN), are thought to be associated with cognitive processes such as introspection, self-monitoring, prospection, episodic and autobiographic memory, and comprehension of the emotional states and intentions of others [7], [8], [9]. The DMN is characterized by positive and negative connectivity between the dorsal and ventral medial prefrontal cortex (MPFC), the medial parietal cortex (posterior and anterior cingulate cortex (PCC; ACC), precuneus (preCUN)), and the inferior parietal cortex during rest [7], [10], [11], [12]. Given the resource enhancing effects of visual arts, we hypothesized that participation in 10-week-long visual art groups may result in psychological changes and may alter the functional interplay of the DMN. Therefore, we used fMRI to investigate the DMN of a non-clinical sample of 28 post-retirement adults before (T0) and after (T1) participating in a visual art production group (8 female, 6 males, mean age 63.50 years ±3.80 SD) or in a cognitive art evaluation group (7 female, 7 males, mean age 63.93 years ±3.34 SD). The participants were randomly assigned to groups, which were stratified by gender and age. The cognitive art evaluation group served as a means of controlling for other possible alternative explanations of changes in the visual art production intervention that may have been related to group interaction, art reception, and cognitive activity, group activity and cognitive activity were thus considered to be present in both intervention groups. Transition into retirement has been found to be associated with well-being, stress experience, and health conditions [13]. The effects of job characteristics and pre-retirement resources on the well-being of retirees are also well documented [14]. Not much is known, however, about the possible effects of post-retirement activity on the stabilization of well-being [15]. Typically, normal ageing is accompanied by changes in brain physiology that involve neural degeneration but by compensatory mechanisms as well [16]. Therefore, we expected that post-retirement adults would be susceptible to the stabilizing effects of receiving artistic training. To understand the psychological relevance of functional changes, we assessed psychological resilience, i.e. stress resistance. Psychological resilience is conceptualized as a protective personality characteristic that allows individuals to control negatives effects of stress and thus enables a successful and healthy functioning even in stressful life conditions [17], [18]. Its neural correlates are thought to be located in the MPFC, which forms a core component of the DMN [19]. To test our results regarding functional connectivity in the DMN, we used the visual cortex (VisCx) as a control site. Due to the pivotal role of the primary sensory and motor cortices (S1/M1) in sensory and motor processing, we also studied the functional connectivity of S1/M1 at rest.

## Materials and Methods

### Experimental Design

From February through April 2011, 28 participants were recruited via advertisements in local newspapers. The study included participants who were between the ages of 62 and 70 (±2 years) and who had been retired for at least 3 months but no longer than 3 years. The participants also needed to have sufficient time available over a period of 10 weeks for attending the art class interventions. Excluded from participation were professional visual artists and art historians, as well as people suffering from serious physical or mental disorders or taking psychotropic drugs. Table 1 shows the epidemiologic data. All participants completed a psychological examination and an fMRI measurement on 2 occasions: at pre-intervention (T0) and at post-intervention 10 weeks later (T1). The psychological examination consisted of the brief German version of the Resilience Scale (RS-11) by Wagnild & Young (1993) which is a valid and reliable instrument for measuring the individuals' capacity of stress resistance in elderly participants [18], [20], [21]. Participants rated their accordance of 11 resilience items on a 7-point Likert scale ranging from 1 (never) to 7 (always). Before the fMRI measurement, all participants were instructed to keep their eyes closed during the scan, to be relaxed but to not fall asleep. After the functional measurement the participants were asked if they followed the instructions. All participants were informed about the procedures of the study and gave informed written consent in line with the Declaration of Helsinki. The study was approved by the local ethics committees of the University of Erlangen-Nuremberg.

**Table 1:** Epidemiologic data.

		Visual art production	Cognitive art evaluation	Total
Number of participants		14	14	28
**Age**		63.50 (±3.80 SD)	63.93 (±3.34 SD)	63.71 (±3.52 SD)
**Sex**	Female	8	7	15
	Male	6	7	13
**Handedness**	Right- handed	11	13	24
	Left- handed	1	1	2
	Ambidextrous	2	0	2
**Education**	Low	5	0	5
	Middle	6	5	11
	High	3	9	12
**Retired since**	0–12 months	9	6	15
	12–24 months	2	3	5
	24–36 months	3	5	8
**Number of attendances**	6 sessions	0	1	1
	7 sessions	2	3	5
	8 sessions	1	3	4
	9 sessions	5	3	8
	10 sessions	6	4	10

### Art Interventions

Two different art interventions took place, each lasting two hours and occurring once a week for 10 weeks in the Germanisches Nationalmuseum and in the rooms of the Art Education Department of the Museums in Nuremberg (Germany). The two interventions were based on different methodological concepts. In the visual art production group the participants actively created art, and in the cognitive art evaluation group the participants cognitively evaluated pieces of art. The concept of visual art production intervention focused on discovering and developing the participant's own creativity. A visual artist trained as an art educator introduced different artistic methods and materials used in drawing and painting, and the participants were then able to experiment with different materials and techniques. Each participant was encouraged to produce visual art and find their own personal form of artistic expression. Each session adhered closely to a precisely defined schedule, which included a sequence of thematic foci such as blind or fast drawing, drawing in the space/room, drawing still lives and figures, drawing with music, using colours, and composition. In the cognitive art evaluation group participants considered, analysed, and interpreted selected paintings and sculptures, in dialogue with a qualified art historian. The art historian helped encourage group discussion by providing expert background information and explaining associations between the work of art and everyday experiences. In each session the pre-defined schedule was strictly adhered to, and participants were required to consider two pieces of art that involved universal human concerns such as age, youth, love, lust, violence, the experience of nature, or faith. Such methodological concepts might improve the art experience [22], [23], [24].

### FMRI Data Acquisition

Echoplanar images were collected on a 3 Tesla MRI scanner (Trio, Siemens Healthcare, Erlangen, Germany) using the standard head coil in the following order: First, a T1-weighted three-dimensional magnetization prepared rapid acquisition gradient-echo sequence (MPRAGE) scan (voxel size  = 1.0×1.0×1.0 mm^3^) was recorded for the individual brain anatomy and lasted 8 min and 21 s. Then, for each participant the time-series of 150 whole-brain images were obtained with a gradient-echo, echo-planar scanning sequence (EPI; TR 3 s, time to echo 40 ms, flip angle 90°; field of view 220×220 mm, acquisition matrix 128×128, 24 axial slices, slice thickness 4 mm, and gap 1 mm). The functional MRI data scan lasted 7 min and 30 s.

### FMRI Data Analysis

Data analysis, registration, and visualization were performed with Brain Voyager QX version 1.10 (Brain Innovation, Maastricht, Netherlands). Data-motion-correction was implemented by the installed software package (Siemens, syngo MR B15) of the scanner. Afterwards, the data were motion corrected using sinc interpolation. Preprocessing also included Gaussian spatial (full width at half maximum (FWHM)  = 4 mm) and temporal (FWHM  = 3 volumes) smoothing of the functional data to reduce artefacts. The functional data were then linear-interpolated to 3.0×3.0×3.0 mm^3^ resolution and transformed into a standard stereoactic coordinate system of Talairach & Tournoux (1988). By using a Talairach daemon after analysis, we were able to locate the relevant brain regions of activations of functional connectivity [25]. Since the removal of the global signal as a further preprocessing step is very controversial, the global signal was included [26]. Anatomical data of each participant were averaged for group analysis. A z-transformation of the functional volume time course for each participant was applied to account for different baseline signal levels. Functional connectivity maps of the DMN, VisCX and S1/M1 were calculated using a seed voxel correlation analysis (SCA). After extracting the individual signal time course of each participant from three defined regions of interests (ROIs, 10×10×10 mm^3^; PCC/preCUN; x = ±6, y = −54, z = 3, S1/M1; x = −32, y = −30, z = 44, VisCx; x = ±6, y = −72, z = 1) the signal time course was correlated with the signal time course of every other brain voxel. Due to the association of hemispheric specialization and creative thinking, the PCC/preCUN coordinates were located in both hemispheres [27]. The PCC/preCUN has been used in previous studies to explore the functional connectivity of the DMN [28], [29], [30]. The coordinates of S1/M1 were determined from an fMRI-study by Maihöfner et al. (2007) [31]. The coordinates of the VisCx were determined from an fMRI-study by Malinen et al. (2010) in which they investigated the functional connectivity in the DMN and used the VisCx to validate their findings [32]. Using the general linear model (GLM) the individual analysis resulted in a t-statistic map. Subsequently, connectivity analysis at the group level was performed. To identify significant group-related differences, group-level contrast maps were calculated between the connectivity maps of the ROIs. Paired-t-tests were used to compare the unsigned connectivity maps of T0 and T1 in each group. Connectivity maps were thresholded at P<0.0001 (after Bonferroni correction, two-tailed) and visualized at 9.5<T>15.5. Group-level contrast maps (T1>T0) were determined by a (q<0.001) FDR corrected threshold. The resulting contrast maps of t-values were visualized at 3.22<T>8.00. In all group analyses, a minimum cluster size of 108 mm^3^ (4 voxels) was applied. The cluster size criterion was used as a conservative measure to minimize false positive activations due to type 1 errors [33]. The corresponding p-values at the cluster level were corrected for multiple comparisons.

### Statistical Analysis

Epidemiological and psychological data were analysed using SPSS v.18.0.0 (SPSS, Inc., Chicago, IL). The epidemiological data are presented as mean ± SD and the psychological data as mean ± SEM. For a comparison of T0 and T1 in each group, we performed Wilcoxon signed-rank tests due to the small sample size in each group. Statistical significance was assumed for P<0.05 (see Text S1 for details). To measure correlations between the functional connectivity of the right and left PCC/preCUN and the psychological resilience, an analysis of covariance (ANCOVA), as implemented in the Brain Voyager, was performed. The resilience score at T1 of the visual art production group served as a covariate of interest. The ANCOVA was performed using a random-effects analysis. Correlation maps were thresholded at P<0.05 (uncorrected) and were visualized at 0.53<T>1.00. At cluster level, values of P<0.05 (uncorrected) were considered to be statistically significant. A minimum cluster size of 108 mm^3^ (4 voxel) was applied which was used as a conservative measure to minimize false positive activations due to type 1 errors [33].

## Results

### Psychological Examination

We found a significant improvement (P = 0.013) in psychological resilience from pre-intervention (T0) (60.64 points ±1.71 SEM) to post-intervention (T1) (63.50 points ±1.47 SEM) in the visual art production group. In the cognitive art evaluation group, in contrast, no significant improvement (P = 0.195) in psychological resilience from T0 (62.57 points ±2.32 SEM) to T1 (64.79 points ±1.80 SEM) was found (see Table 2).

**Table 2:** Psychological resilience.

Group	n	Pre-intervention	Post-intervention	P-value
Visual art production	14	60.64 (±1.71 SEM)	63.50 (±1.47 SEM)	0.013*
Cognitive art evaluation	14	62.57 (±2.32 SEM)	64.79 (±1.80 SEM)	0.195

*significant at 0.05.

### Default Mode Network (DMN)

**Figure 1:**
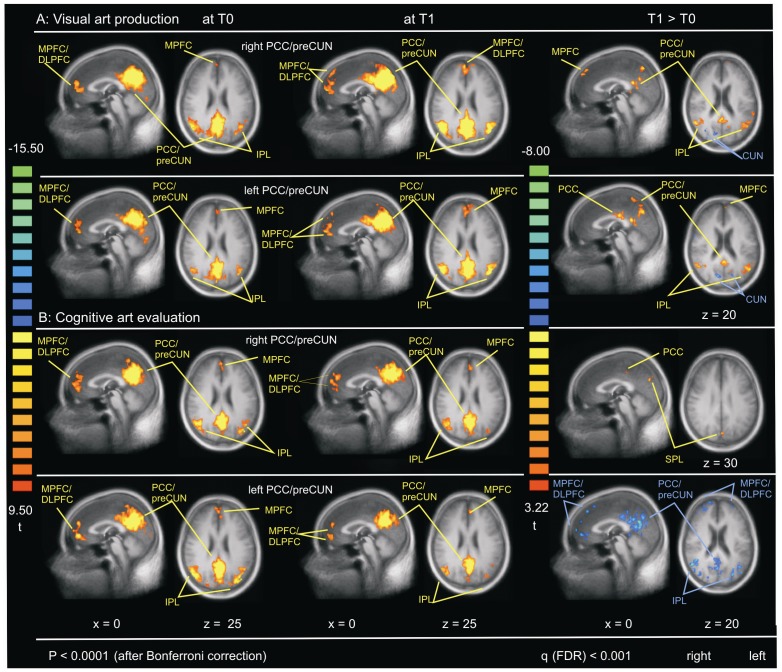
The default mode network (DMN). Brain regions that show significant functional connectivity of the PCC/preCUN in: (**A**) visual art production group (**B**) cognitive art evaluation group at pre-intervention (T0), post-intervention at 10 weeks (T1), and contrast T1 (red) >T0 (blue). PCC/preCUN used in each group is shown on the left and right sides.

For the identification of the DMN by a seed voxel correlation analysis (SCA), we chose a region of interest (ROI) in the PCC/preCUN as described in the methods. Figure 1 shows brain areas with significant functional connectivity to the right and left PCC/preCUN at T0 and T1 (see Table S1 for details). Generally, we were able to identify the DMN in the visual art production group at both points in time (see Figure 1A). The common brain areas included the frontal cortices (BA 6, 8, 9, 10, 45, and 46) with extension across the middle and superior temporal gyri (MTG, BA 21; STG, 22) to the inferior parietal lobule (IPL, BA 39; PCC, BA 31) in both hemispheres. At T1 several DMN areas showed significant bilateral increases in functional connectivity of the left and right PCC/preCUN to the premotor cortex (BA 6), prefrontal cortex (BA 8, 9, 10, 46), to the superior and inferior parietal lobules (SPL, BA 7; IPL, BA 39, 40), PCC (BA 23, 30, 31), and to the MTG and STG (BA 21, BA 22) compared to T0. The cognitive art evaluation group presented similar findings at T0 as the visual art production group (see Figure 1B). At T1, however, the cognitive art evaluation group had only significantly greater functional connectivity from the right PCC/preCUN to SPL (BA 7) and to PCC (BA 31) compared to T0. No improvements were found for the left PCC/preCUN at T1 compared to T0.

### Visual Cortex (VisCx)

**Figure 2:**
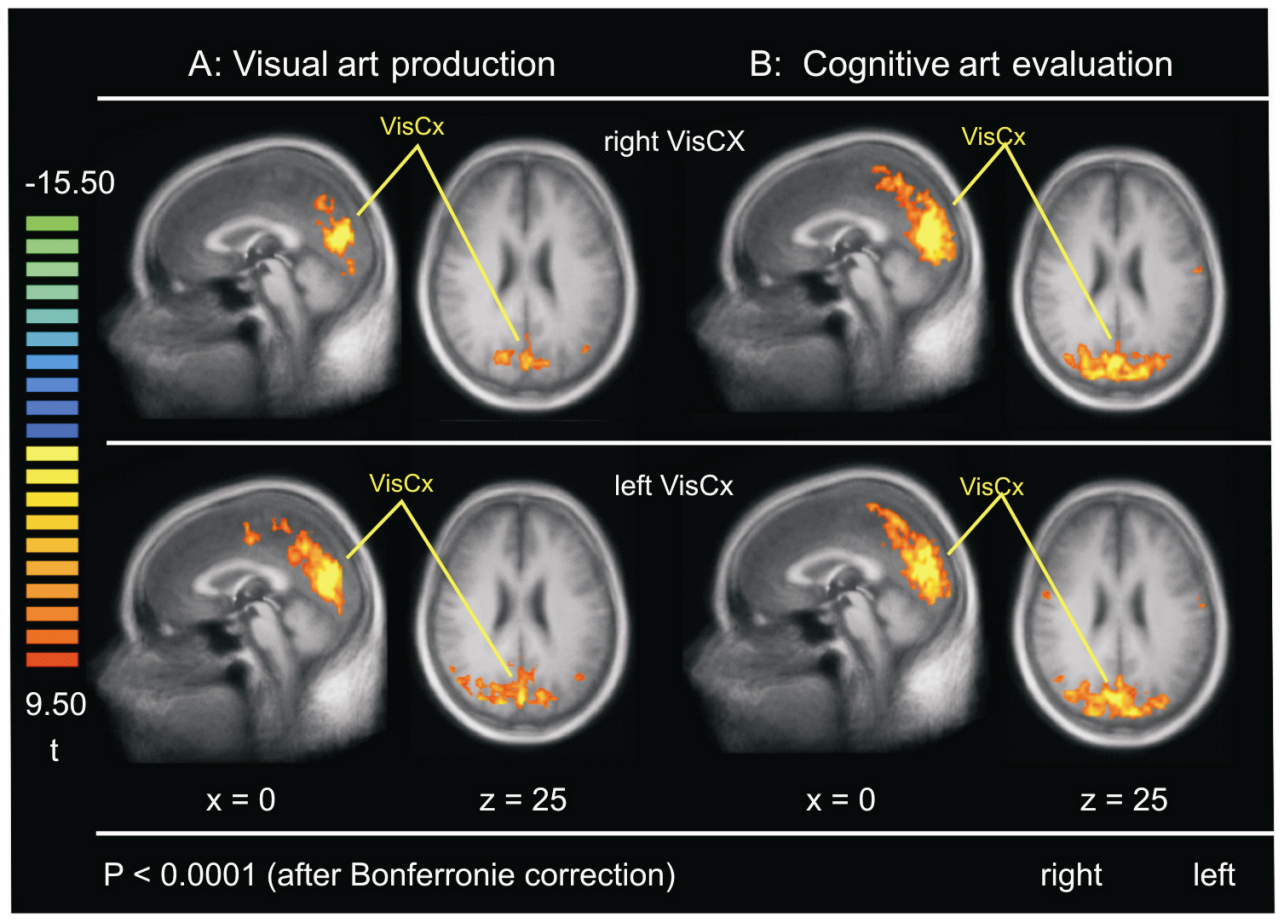
The visual cortex (VisCx). Brain regions that show significant functional connectivity of VisCx in: (**A**) visual art production group (**B**) cognitive art evaluation group. VisCx used in each group is shown on the left and right sides.

To validate our findings regarding the functional connectivity of the DMN, we chose a ROI in the visual cortex. The functional connectivity of VisCx at rest included the occipital and parietal cortices. No functional connectivity pattern like the DMN was observed in the visual art production group or in the cognitive art evaluation group (see Figure 2 and Table S2 for details).

### Sensorimotor Cortex (S1/M1)

**Figure 3:**
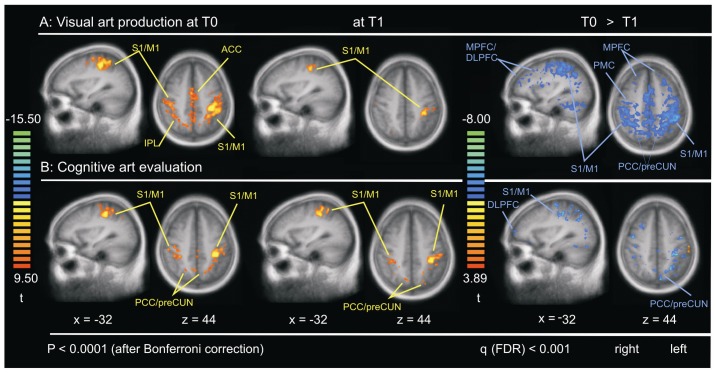
The sensorimotor cortex (S1/M1). Brain regions that show significant functional connectivity of S1/M1 in: (**A**) visual art production group (**B**) cognitive art evaluation group at pre-intervention (T0), post-intervention at 10 weeks (T1), and contrast T0 (blue) >T1 (red). S1/M1 used in each group is shown on the left side.

For our investigation of the functional connectivity of the sensorimotor cortex at rest, a ROI around the S1/M1 was chosen. Due to the majority of right-handed participants (85.71%), we located the ROI in the left hemisphere. Handedness was measured with the German version of the Edinburgh Handedness Inventory (see Table 1) [34]. In both groups at T0 the SCA showed intraregional connectivity within S1/M1 with bilateral diffuse functional connectivity to the cingulate cortex, to the right S1/M1, and to the IPL. At T1 both groups showed significantly stronger intraregional connectivity of S1/M1 and less connectivity with other regions compared to T0 (see Figure 3 and Table S3 for details).

### DMN and Resilience

**Figure 4:**
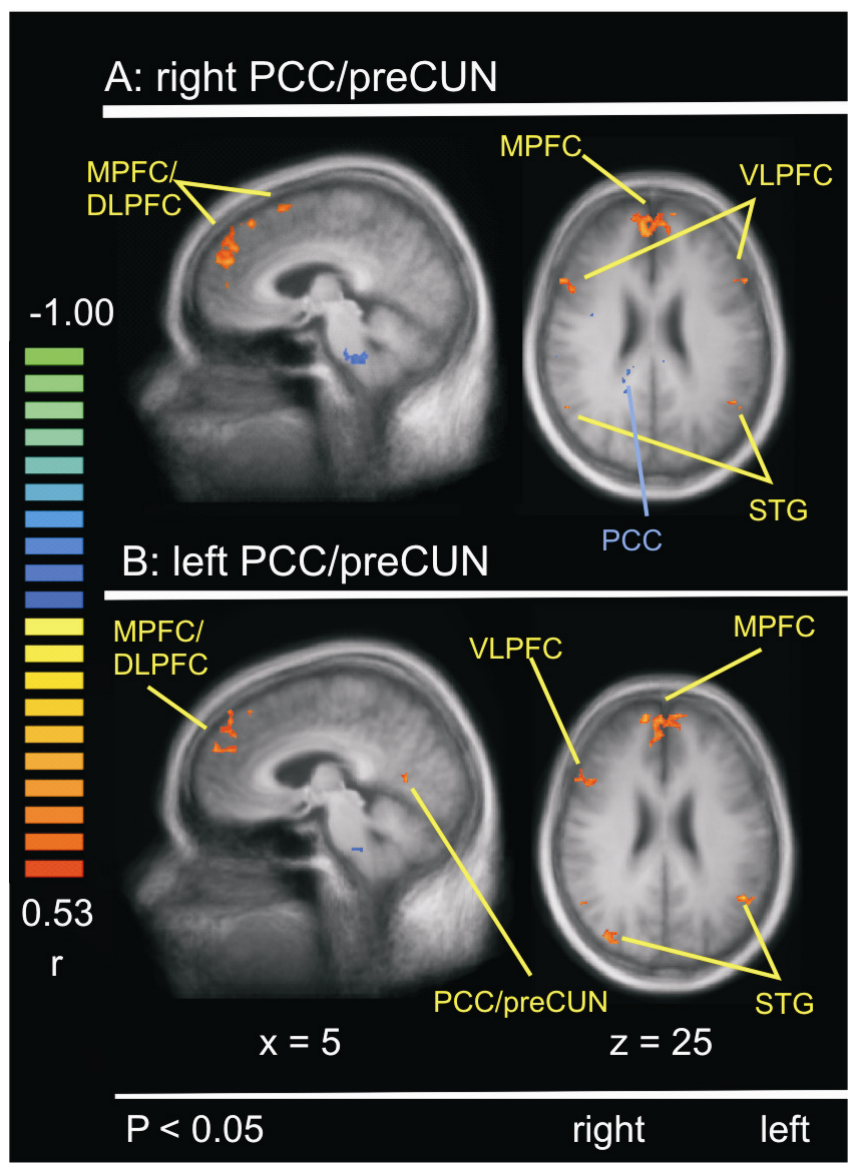
Analysis of covariance (ANCOVA). Covariations between DMN and resilience in the visual art production group at post-intervention (T1). Red – positive correlation; Blue – negative correlation. (**A**) Covariation between right PCC/preCUN and resilience at T1 (**B**) Covariation between left PCC/preCUN and resilience at T1.

In order to link the psychological resilience with the functional connectivity of the DMN in the visual art production group, we applied an ANCOVA (see Figure 4 and Table S4 for details). A statistically significant correlation (greater resilience in relation to greater PCC/preCUN functional connectivity) was noted for the frontal cortices (BA 6, r = 0.60; BA 8, r = 0.62; BA 9, r = 0.63; BA 10, r = 0.60; BA 45, r = 0.60; BA 47, r = 0.60). In addition, we found that greater resilience was associated with greater functional connectivity of the DMN with STG and MTG (BA 22, r = 0.63; BA 21, r = 0.59). In contrast, a negative correlation (greater resilience in relation to less DMN functional connectivity) was observed for the parietal cortex (BA 40, r = −0.60; BA 31, r = −0.58; BA 23, r = −0.57).

## Discussion

In the current study we used fMRI to investigate whether visual art production and cognitive art evaluation had different effects on the functional interplay of the DMN in a non-clinical sample of 28 post-retirement adults. Our findings demonstrate that training in a visual art production group enhances functional connectivity of the DMN, particularly between the parietal and frontal cortices. No such effects were observed in a cognitive art evaluation intervention group.

### DMN

Recent fMRI studies on various neuropsychiatric disorders [35], [36], [37] as well as on chronic pain have demonstrated disruptions in the temporal and spatial properties of functional connectivity at rest [32], [38], [39]. Moreover, normal ageing also seems to affect the intrinsic activity and connectivity of the DMN [29], [40], [41], [42], [43], [44]. Age-related alterations in the DMN are proposed to result in less effective functional interactions between brain regions. Damoiseaux & colleagues (2008), for example, found that non-clinical samples of older participants (compared to younger participants) had less connectivity between the superior and middle frontal cortex, PCC, and the superior parietal gyrus [44]. Consistent with this finding, Sambataro & colleagues (2010) observed an age-related reduction in the connectivity between PCC and the MPFC, which was correlated with poor working memory performance [40]. Our study also included an older non-clinical sample and showed particular improvement in the connectivity of the PCC/preCUN to the bilateral frontal cortices after producing visual art.

In contrast, no hard evidence of improved connectivity derives from the cognitive art evaluation group. The functional connectivity of the right PCC/preCUN showed only weak improvements between T0 and T1, and the left PCC/preCUN showed no change at all. The improvements included stronger connectivity to SPL and PCC. Generally, the evaluative reception of artwork is an aesthetic experience, in which the parietal regions of the brain, especially the SPL, are associated with visuo-spatial exploration and attention [45]. Furthermore, the improvements of only the right PCC/preCUN may result from hemispheric specialization in creative activities. Despite distinct empirical evidence, a recent meta-analysis by Mihov et al. (2010) demonstrated a relative dominance of the right hemisphere during abstract thinking, i.e., creative thinking, which was required in the cognitive art evaluation group [27]. Generally, functional studies suggest that the parietal and frontal cortices play key roles in creative activities [46]. It should be considered that the cognitive art evaluation group had higher education levels than the visual art production group. One could argue that this fact might contribute to possible habituations effects. However, as stated in the method section, we included participants who had no previous education in visual art and were no professional visual artists or art historians. Consequently, the participants had all the same starting point in contact with visual art at the beginning of the interventions.

The question of why the two groups demonstrated different changes in functional connectivity at rest remains open. The improvements in the visual art production group may be partially attributable to a combination of motor and cognitive processing. Other recent fMRI studies have demonstrated enhancements in the functional connectivity between the frontal, posterior, and temporal cortices after the combination of physical exercises and cognitive training [42], [47]. The participants in our study were required to perform the cognitive tasks of following, understanding, and imitating the visual artist's introduction. Simultaneously, the participants had to find an individual mode of artistic expression and maintain attention while performing their activity. Although we cannot provide mechanistic explanations, the production of visual art involves more than the mere cognitive and motor processing described. The creation of visual art is a personal integrative experience - an experience of “flow,” - in which the participant is fully emerged in the creative activity [48].

### Resilience and Functional Connectivity

The psychological relevance of the reported findings is illustrated by the statistically significant correlation between functional connectivity of PCC/preCUN in the prefrontal lobes and psychological resilience at T1 in the visual art production group. In general, regions of the prefrontal cortex, particularly BA 8, 9, and 10, are robustly activated during introspection [8]. Several fMRI studies have shown that MPFC activations are associated with the use of cognitive strategies to reduce negative emotional experience – suggesting that the MPFC is responsible for the successful cognitive regulation of emotions [49]. Moreover, increased activation of the anterior MPFC is correlated with a greater self-awareness, as a recent fMRI study by Jang and colleagues has demonstrated [50]. Our findings also point to greater activations and correlations to the anterior MPFC. This may indicate increased self-awareness, as a result of the methodological approaches applied in the visual art production intervention. The visual art production intervention involved the development of personal expression and attentional focus on self-related experience during art creation. Another interesting finding is a statistically significant correlation between resilience and functional connectivity of the PCC/preCUN in MTG and STG at T1. The medial temporal lobes play a central role in memory processing [51]. Thus, such associations point to enhanced memory processing, which is indeed required when stored knowledge is connected with new information to produce creative works [52]. Moreover, the lack of significant improvement in resilience in the cognitive art evaluation group strengthens the suggestion that visual art production has an impact on psychological resilience.

### Sensorimotor Cortex

We found that the visual art production group at T1 had significantly stronger intraregional connectivity in S1/M1 and less connectivity with other brain regions compared to T0. The cognitive art evaluation group also showed stronger interregional connectivity at T1. However, the changes were less strong than in the visual art production group. Obviously, the increased intraregional connectivity demonstrates an improved specificity and differentiation of S1/M1 at rest. The loss of specialization in certain regions of the brain, with reduced distinctiveness or differentiation at the neural level, is generally thought to represent a compensational strategy of the ageing brain [42], [53]. Furthermore, our results strengthen the suggestion that the changes observed in functional connectivity were induced by the intervention.

### Prospect

Our findings imply that the production of visual art improves effective interaction between brain regions of the DMN and increases the specificity and differentiation of S1/M1 at rest. Moreover, the improvements are associated with better resilience scores, meaning that our results may have important implications for preventive and therapeutic interventions. By the year 2030 one-fifth of Americans will be 65 or older, which will mean a greater number of challenging health conditions [54]. Our results revealed that visual art production leads to improved interaction, particularly between the frontal and posterior and temporal brain regions, and thus may become an important prevention tool in managing the burden of chronic diseases in older adults. In the context of therapeutic intervention, further research is required to investigate whether improvements in disrupted functional connectivity of the DMN are associated with positive consequences for cognitive, emotional, and behavioural functions in various clinical disorders.

## Supporting Information

Table S1 **Regions of functional connectivity depicted in** **Fig. 1** **.**
(DOC)

Table S2 **Regions of functional connectivity depicted in** **Fig. 2** **.**
(DOC)

Table S3 **Regions of functional connectivity at rest depicted in** **Fig. 3** **.**
(DOC)

Table S4 **Correlation between functional connectivity and resilience depicted in** **Fig. 4** **.**
(DOC)

Text S1 **Estimation of missing data with an analysis of regression.**
(DOC)

## References

[1] Stuckey HL, Nobel J (2010) The Connection Between Art, Healing, and Public Health: A Review of Current Literature. Am J Public Health 100: 254–263. 20019311 10.2105/AJPH.2008.156497 PMC2804629

[2] Cohen GD, Perlstein S, Chaplin J, Kelly J, Firth KM, et al (2006) The Impact of Professionally Conducted Cultural Programs on the Physical Health, Mental Health, and Social Functioning of Older Adults. Gerontologist 46: 726–734. 17169928 10.1093/geront/46.6.726

[3] Leckey J (2011) The therapeutic effectiveness of creative activities on mental well-being: a systematic review of the literature. J Psychiatr Ment Health Nurs 18: 501–509. 21749556 10.1111/j.1365-2850.2011.01693.x

[4] Geue K, Goetze H, Buttstaedt M, Kleinert E, Richter D, et al (2010) An overview of art therapy interventions for cancer patients and the results of research. Complement Ther Med 18: 160–170. 20688262 10.1016/j.ctim.2010.04.001

[5] Clow A, Fredhoi C (2006) Normalisation of salivary cortisol levels and self-report stress by a brief lunchtime visit to an art gallery by London City workers. J Holist Health 3: 29–32.

[6] Lacey S, Hagtvedt H, Patrick VM, Anderson A, Stilla R, et al (2011) Art for reward's sake: Visual art recruits the ventral striatum. Neuroimage 55: 420–433. 21111833 10.1016/j.neuroimage.2010.11.027 PMC3031763

[7] Buckner RL, Vincent JL (2007) Unrest at rest: Default activity and spontaneous network correlations. Neuroimage 37: 1091–1096. 17368915 10.1016/j.neuroimage.2007.01.010

[8] Gusnard DA, Akbudak E, Shulman GL, Raichle ME (2001) Medial prefrontal cortex and self-referential mental activity: Relation to a default mode of brain function. PNAS 98: 4259–4264. 11259662 10.1073/pnas.071043098 PMC31213

[9] Raichle ME (2006) The Brain's Dark Energy. Science 314: 1249–1250. 17124311

[10] Fox MD, Raichle ME (2007) Spontaneous fluctuations in brain activity observed with functional magnetic resonance imaging. Nat Rev Neurosci 8: 700–711. 17704812 10.1038/nrn2201

[11] Raichle ME, MacLeod AM, Snyder AZ, Powers WJ, Gusnard DA, et al (2001) A default mode of brain function. PNAS 98: 676–682. 11209064 10.1073/pnas.98.2.676 PMC14647

[12] Damoiseaux JS, Rombouts SARB, Barkhof F, Scheltens P, Stam CJ, et al (2006) Consistent resting-state networks across healthy subjects. PNAS 103: 13848–13853. 16945915 10.1073/pnas.0601417103 PMC1564249

[13] Fehr R (2012) Is retirement always stressful? The potential impact of creativity. Am Psychol 67: 76–77. 22229631 10.1037/a0026574

[14] Herzog RA, House JS, Morgan JN (1991) Relation of work and retirement to health and well-being in older age. Psychol Aging 6: 202–211. 1863389 10.1037//0882-7974.6.2.202

[15] Marshall VW, Clarke PJ, Ballantyne PJ (2001) Instability in the Retirement Transition: Effects on Health and Well-Being in a Canadian Study. Res Aging 23: 379–409.

[16] Goh JO, Park DC (2009) Neuroplasticity and cognitive aging: The scaffolding theory of aging and cognition. Restor Neurol Neurosci 27: 391–403. 19847066 10.3233/RNN-2009-0493 PMC3355626

[17] Windle G (2010) The Resilience Network. What is resilience? A systematic review and concept analysis. Rev Clin Geronto 21: 1–18.

[18] Wagnild GM, Young HM (1993) Development and psychometric evaluation of the Resilience Scale. J Nurs Meas 1: 165–178. 7850498

[19] Maier SF, Watkins LR (2010) Role of the medial prefrontal cortex in coping and resilience. Brain Res 1355: 52–60. 20727864 10.1016/j.brainres.2010.08.039 PMC2967290

[20] Schumacher J, Leppert K, Gunzelmann T, Strauβ B, Brähler E (2005) The Resilience Scale - A questionnaire to assess resilience as a personality characteristic. Die Resilienzskala - Ein Fragebogen zur Erfassung der psychischen Widerstandsfähigkeit als Personmerkmal. Z Klin Psychol Psychiatr Psychother 53: 16–39.

[21] Leppert K, Gunzelmann T, Schuhmacher J, Strauss B, Brähler E (2005) Resilience as a protective personality characteristic in the elderly. Psychother Psychosom Med Psychol 55: 365–9. 16049872 10.1055/s-2005-866873

[22] Packer J (2008) Beyond Learning: Exploring Visitors' Perception of the Value and Benefits of Museum Experience. Curator 51: 33–54.

[23] Silverman LH (2010) The Social Work of Museums, London and New York: Routledge.

[24] Peez G (2002) Qualitative empirische Forschung in der Kunstpädagogik. Methodologische Analysen und praxisbezogene Konzepte zu Fallstudien über ästhetische Prozesse, biografische Aspekte und soziale Interaktion in unterschiedlichen Bereichen der Kunstpädagogik.Norderstedt: Books on Demand.

[25] Talairach J, Tournoux P (1988) Co-Planar Stereotaxic Atlas of the Human Brain. New York: Thieme Medical Publishers.

[26] Murphy K, Birn RM, Handwerker DA, Jones TB, Bandettini PA (2009) The impact of global signal regression on resting state correlations: are anti-correlated networks introduced? Neuroimage 44: 893–905. 18976716 10.1016/j.neuroimage.2008.09.036 PMC2750906

[27] Mihov KM, Denzler M, Förster J (2010) Hemispheric specialization and creative thinking: A meta-analytic review of lateralization of creativity. Brain Cog 72: 442–448. 10.1016/j.bandc.2009.12.007 20097463

[28] Pyka M, Burgmer M, Lenzen T, Pioch R, Dannlowski U, et al (2011) Brain correlates of hypnotic paralysis - a resting-state fMRI study. Neuroimage 56: 2173–2182. 21497656 10.1016/j.neuroimage.2011.03.078

[29] Andrews-Hanna JR, Snyder AZ, Vincent JL, Lustig C, Head D, et al (2007) Disruption of Large-Scale Brain Systems in Advanced Aging. Neuron 56: 924–935. 18054866 10.1016/j.neuron.2007.10.038 PMC2709284

[30] Grady CL, Protzner AB, Kovacevic N, Strother SC, Afshin-Pour B, et al (2010) A Multivariate Analysis of Age-Related Differences in Default Mode and Task-Positive Networks across Multiple Cognitive Domains. Cereb Cortex 20: 1432–1447. 19789183 10.1093/cercor/bhp207 PMC3181214

[31] Maihöfner C, Baron R, De Col R, Binder A, Birklein F, et al (2007) The motor system shows adaptive changes in complex regional pain syndrome. Brain 130: 2671–2687. 17575278 10.1093/brain/awm131

[32] Malinen S, Vartiainen N, Hlushchuk Y, Koskinen M, Ramkumar P, et al (2010) Aberrant temporal and spatial brain activity during rest in patients with chronic pain. PNAS 107: 6493–6497. 20308545 10.1073/pnas.1001504107 PMC2852014

[33] Maihöfner C, Handwerker HO (2005) Differential coding of hyperalgesia in the human brain: A functional MRI study. Neuroimage 28: 996–1006. 16112876 10.1016/j.neuroimage.2005.06.049

[34] Oldfield RC (1971) The assessment and analysis of handedness: the Edingburg inventory. Neuropsychologia 9: 97–113. 5146491 10.1016/0028-3932(71)90067-4

[35] Williamson P (2007) Are Anticorrelated Networks in the Brain Relevant to Schizophrenia? Schizophr Bull 33: 994–1003. 17493957 10.1093/schbul/sbm043 PMC2632338

[36] Kennedy DP, Redcay E, Courchesne E (2006) Failing to deactivate: Resting functional abnormalities in autism. PNAS 103: 8275–8280. 16702548 10.1073/pnas.0600674103 PMC1472462

[37] Greicius MD, Flores BH, Menon V, Glover GH, Solvason HB, et al (2007) Resting-State Functional Connectivity in Major Depression: Abnormally Increased Contributions from Subgenual Cingulate Cortex and Thalamus. Biol Psychiatry 62: 429–437. 17210143 10.1016/j.biopsych.2006.09.020 PMC2001244

[38] Baliki MN, Geha PY, Apkarian AV, Chialvo DR (2008) Beyond Feeling: Chronic Pain Hurts the Brain, Disrupting the Default-Mode Network Dynamics. J, Neurosci 28: 1398–1403. 18256259 10.1523/JNEUROSCI.4123-07.2008 PMC6671589

[39] Cauda F, Sacco K, Duca S, Cocito D, D'Agata F, et al (2009) Altered Resting State in Diabetic Neuropathic Pain. PLoS ONE 4: e4542. 19229326 10.1371/journal.pone.0004542 PMC2638013

[40] Sambataro F, Murty VP, Callicott JH, Tan H-Y, Das S, et al (2010) Age-related alterations in default mode network: Impact on working memory performance. Neurobiol Aging 31: 839–852. 18674847 10.1016/j.neurobiolaging.2008.05.022 PMC2842461

[41] Hampson M, Driesen NR, Skudlarski P, Gore JC, Constable RT (2006) Brain Connectivity Related to Working Memory Performance. J Neurosci 26: 13338–13343. 17182784 10.1523/JNEUROSCI.3408-06.2006 PMC2677699

[42] Voss MW, Prakash RS, Erickson KI, Basak C, Chaddock L, et al (2010) Plasticity of brain networks in a randomized intervention trial of exercise training in older adults. Front Aging Neurosci 2: 1–7. 20890449 10.3389/fnagi.2010.00032 PMC2947936

[43] Wu J-T, Wu H-Z, Yang C-G, Chen W-X, Zhang H-Y, et al (2011) Aging-related changes in the default mode network and its anti-correlated networks: A resting-state fMRI study. Neurosci Lett 504: 62–67. 21925236 10.1016/j.neulet.2011.08.059

[44] Damoiseaux JS, Beckamnn CF, Arigita EJS, Barkhof F, Scheltens P, et al (2008) Reduced resting-state brain activity in the “default network” in normal aging. Cereb Cortex 18: 1856–1864. 18063564 10.1093/cercor/bhm207

[45] Corbetta M, Shulman GL (2002) Control of goal-directed and stimulus-driven attention in the brain. Nat Rev Neurosci 3: 201–215. 11994752 10.1038/nrn755

[46] Jung RE, Segall JM, Jeremy Bockholt H, Flores RA, Smith SM, et al (2010) Neuroanatomy of creativity. Hum Brain Mapp 31: 398–409. 19722171 10.1002/hbm.20874 PMC2826582

[47] Voss MW, Erickson KI, Prakash RS, Chaddock L, Malkowski E, et al (2010) Functional connectivity: A source of variance in the association between cardiorespiratory fitness and cognition? Neuropsychologia 48: 1394–1406. 20079755 10.1016/j.neuropsychologia.2010.01.005 PMC3708614

[48] Csikszentmihalyi M (1996) Creativity, flow and the psychology of discovery and invention. New York: Harper Perennial.

[49] Wager TD, Davidson ML, Hughes BL, Lindquist MA, Ochsner KN (2008) Prefrontal-Subcortical Pathways Mediating Successful Emotion Regulation. Neuron 59: 1037–1050. 18817740 10.1016/j.neuron.2008.09.006 PMC2742320

[50] Jang JH, Jung WH, Kang D-H, Byun MS, Kwon SJ, et al (2011) Increased default mode network connectivity associated with meditation. Neurosci Lett 487: 358–362. 21034792 10.1016/j.neulet.2010.10.056

[51] Eichenbaum H, Yonelinas AP, Ranganath C (2007) The Medial Temporal Lobe and Recognition Memory. Annu Rev Neurosci 30: 123–152. 17417939 10.1146/annurev.neuro.30.051606.094328 PMC2064941

[52] Fink A, Benedek M, Grabner RH, Staudt B, Neubauer AC (2007) Creativity meets neuroscience: Experimental tasks for the neuroscientific study of creative thinking. Methods 42: 68–76. 17434417 10.1016/j.ymeth.2006.12.001

[53] Park DC, Reuter-Lorenz P (2009) The Adaptive Brain: Aging and Neurocognitive Scaffolding. Annu Rev Psychol 60: 173–196. 19035823 10.1146/annurev.psych.59.103006.093656 PMC3359129

[54] He W, Sengupta M, Velkoff VA, DeBarros KA (2005) 65+ in the United States: 2005. Current Population Reports. Washington (DC): US Department of Commerce/US Department of Health and Human Services.

