# Correction: How Art Changes Your Brain: Differential Effects of Visual Art Production and Cognitive Art Evaluation on Functional Brain Connectivity

**DOI:** 10.1371/journal.pone.0116548

**Published:** 2014-12-22

**Authors:** The PLOS ONE Staff

Table S2, Table S3, and Table S4 are incorrect. Please view the correct Supporting Information tables below.

## Supporting Information

Table S2 **Regions of functional connectivity depicted in Fig. 2.**
(DOC)

Table S3 **Regions of functional connectivity at rest depicted in Fig. 3.**
(DOC)

Table S4 **Correlation between functional connectivity and resilience depicted in Fig. 4.**
(DOC)
