# Supplementary material for: How Art Changes Your Brain: Differential Effects of Visual Art Production and Cognitive Art Evaluation on Functional Brain Connectivity
Source: PLoS One. 2014 Jul 1;9(7):e101035. doi: 10.1371/journal.pone.0101035 (PMC4077746; doi:10.1371/journal.pone.0101035)
Supplement: Table S1 — Regions of functional connectivity depicted in Fig. 1 . (DOC) [file pone.0101035.s001.doc]

**Supplementary Table 1**

**Table S1: Regions of functional connectivity depicted in Fig. 1**

**Region Side X Y Z BA t-score *P*-value (corr.) size (mm³)**

**A 1: Visual art production group: right PCC/preCUN at pre-intervention (T0)**

MPFC/DLFfC left 5 52 15 9 19,039 < 0.0001 1861

MPFC right 22 32 49 8 20,239 < 0.0001 769

MPFC right 41 18 41 8 14,845 < 0.0001 145

PMC left -20 27 54 6 14,236 < 0.0001 251

PMC right 35 4 55 6 14,631 < 0.0001 151

VPFC right 21 54 23 10 17,844 < 0.0001 252

VPFC right 27 57 13 10 14,864 < 0.0001 191

VPFC left -2 52 12 10 16,057 < 0.0001 1251

IPL right 44 -55 24 39 35,137 < 0.0001 12002

IPL left -41 -60 24 39 25,774 < 0.0001 4922

PCC right -5 -57 27 31 39,975 < 0.0001 19117

PCC left 6 -58 28 31 44,541 < 0.0001 21202

MTG right 58 -36 -2 21 21,372 < 0.0001 2068

MTG left -59 -39 1 21 20,501 < 0.0001 1444

**A 2: Visual art production group: left PCC/preCUN at pre-intervention (T0)**

MPFC left -22 27 50 8 18,149 < 0.0001 314

MPFC/DLPFC left -3 52 28 9 16,086 < 0.0001 334

VPFC left -2 54 9 10 14,661 < 0.0001 510

VPFC right 2 54 12 10 15,933 < 0.0001 547

VLPFC left -53 21 17 45 16,408 < 0.0001 446

IPL right 38 -60 32 39 21,661 < 0.0001 1838

IPL left -31 -63 31 39 26,901 < 0.0001 3628

PCC left -2 -23 38 24 17,414 < 0.0001 149

PreCUN right 6 -60 27 31 34,520 < 0.0001 12493

PreCUN left -6 -60 26 31 43,406 < 0.0001 18297

CUN right 3 -70 7 30 25,131 < 0.0001 3698

STG right 47 -58 20 39 19,376 < 0.0001 1344

STG left -48 -53 22 39 23,899 < 0.0001 1559

MTG right 61 -32 -2 21 15,631 < 0.0001 416

MTG left -60 -32 -3 21 23,760 < 0.0001 3495

MTG right 58 -35 -10 21 14,833 < 0.0001 144

**B 1: Visual art production group: right PCC/preCUN at post-intervention (T1)**

PMC right 19 28 51 6 23,622 < 0.0001 1841

PMC left -1 -15 68 6 10,126 < 0.0001 124

PMC right 2 -32 67 6 11,671 < 0.0001 137

MPFC right 40 18 43 8 22,806 < 0.0001 1867

MPFC left -20 28 51 8 13,077 < 0.0001 180

MPFC right 17 48 39 8 16,900 < 0.0001 479

MPFC left -2 48 42 8 15,434 < 0.0001 967

MPFC right 5 47 43 8 14,769 < 0.0001 400

MPFC/DLPFC left -40 14 41 9 17,687 < 0.0001 598

MPFC/DLPFC right 7 54 26 9 23,734 < 0.0001 1855

MPFC/DLPFC left -3 53 26 9 21,066 < 0.0001 2764

MPFC/DLPFC right 4 52 14 9 19,952 < 0.0001 917

VPFC right 27 60 10 10 17,125 < 0.0001 783

VPFC left -2 55 12 10 19,368 < 0.0001 1624

VLPFC right 49 26 3 45 13,243 < 0.0001 214

PCC left -6 -51 6 30 23,219 < 0.0001 1706

PCC right 6 -55 29 31 45,217 < 0.0001 17819

PCC left -6 -54 30 31 43,187 < 0.0001 22588

RSCC right 6 -48 7 29 23,181 < 0.0001 1646

PreCUN left -15 -71 29 31 20,574 < 0.0001 1201

PreCUN right 1 -54 51 7 20,252 < 0.0001 2268

PreCUN left -5 -54 53 7 18,696 < 0.0001 1686

CUN right 16 -72 34 7 18,245 < 0.0001 1208

SPL left -3 -38 61 5 15,315 < 0.0001 730

IPL right 42 -56 28 39 37,689 < 0.0001 14031

**Region Side X Y Z BA t-score *P*-value (corr.) size (mm³)**

IPL left -43 -56 24 39 29,796 < 0.0001 10665

MTG right 59 -23 -6 21 28,362 < 0.0001 7404

MTG left -60 -23 -4 21 25,642 < 0.0001 5631

**B 2: Visual art production group: left PCC/preCUN at post-intervention (T1)**

MPFC left -20 27 49 8 17,240 < 0.0001 899

MPFC right 23 31 44 8 12,497 < 0.0001 313

MPFC right 18 47 39 8 10,435 < 0.0001 257

PMC left -38 14 45 6 13,719 < 0.0001 1097

MPFC/DLPFC left -2 53 18 9 21,977 < 0.0001 2632

MPFC/DLPFC left -20 45 34 9 12,286 < 0.0001 485

MPFC/DLPFC right 4 53 20 9 21,290 < 0.0001 1427

VPFC left -28 60 16 10 8,520 < 0.0001 129

VPFC right 27 58 9 10 15,570 < 0.0001 284

PCC right 4 -39 7 29 14,530 < 0.0001 106

PCC left -8 -51 6 30 19,999 < 0.0001 720

PCC right 6 -52 7 30 20,724 < 0.0001 682

PreCUN left -5 -52 30 31 43,285 < 0.0001 21846

PreCUN right 7 -56 27 31 35,417 < 0.0001 8414

SPL right 21 -72 40 7 12,762 < 0.0001 112

IPL left -43 -57 25 39 31,690 < 0.0001 8675

IPL right 43 -56 27 39 30,277 < 0.0001 8630

MTG right 62 -29 -3 21 18,849 < 0.0001 955

MTG left -61 -27 -4 21 16,680 < 0.0001 3566

**C 1: Contrast T0 vs. T1: right PCC/preCUN**

PMC right 13 20 58 6 6,530 < 0.0001 188

MPFC/DLPFC right 45 22 37 9 7,174 < 0.0001 191

MPFC/DLPFC right 3 54 37 9 8,279 < 0.0001 591

MPFC/DLPFC left -28 50 31 9 -5,721 < 0.0001 117

MPFC right 19 45 40 8 6,717 < 0.0001 137

SPL left -5 -49 50 7 3,734 < 0.0001 1002

SPL left -4 -60 41 7 3,010 < 0.0001 432

VPFC left -11 62 34 10 5,867 < 0.0001 151

VPFC right 30 66 9 10 5,886 < 0.0001 212

VPFC left -28 63 3 10 6,122 < 0.0001 184

MTG right 56 -3 -19 21 8,222 < 0.0001 176

MTG left -62 -12 -18 21 7,349 < 0.0001 146

STG right 63 -20 -2 22 10,978 < 0.0001 1497

STG left -61 -34 17 42 5,753 < 0.0001 440

IPL left -46 -56 21 39 7,309 < 0.0001 2236

IPL right 39 -57 34 39 8,001 < 0.0001 4277

IPL left -41 -52 42 40 3,942 < 0.0001 401

IPL right 51 -53 22 39 9,309 < 0.0001 646

PreCUN left -8 -64 25 31 7,907 < 0.0001 556

PreCUN right 2 -64 48 7 4,655 < 0.0001 324

CUN right 19 -73 8 30 -7,905 < 0.0001 244

CUN right 14 -74 19 30 -6,447 < 0.0001 150

PCC right 5 -36 5 30 7,724 < 0.0001 196

PCC left -2 -21 31 23 5,332 < 0.0001 57

PCC right 4 -48 22 23 7,593 < 0.0001 728

PCC left -6 -50 23 23 6,844 < 0.0001 1343

PCC right 8 -52 7 30 4,229 < 0.0001 248

**C 2: Contrast T0 vs. T1: left PCC/preCUN**

PMC left -14 25 55 6 6,997 < 0.0001 129

MPFC left -27 26 40 8 7,781 < 0.0001 212

VPFC right 28 65 8 10 5,985 < 0.0001 162

VPFC left -27 61 1 10 6,881 < 0.0001 402

VPFC right 26 55 7 10 6,408 < 0.0001 143

VPFC left -3 62 16 10 6,372 < 0.0001 161

MPFC/DLPFC right 42 41 31 9 -7683 < 0.0001 186

DLPFC right 52 35 11 46 7,045 < 0.0001 155

IPL right 37 -60 41 39 10,317 < 0.0001 1065

IPL left -45 -56 22 39 9,188 < 0.0001 3501

IPL right 44 -56 29 39 10,299 < 0.0001 3246

**Region Side X Y Z BA t-score *P*-value (corr.) size (mm³)**

IPL right 54 -38 28 40 -6,271 < 0.0001 280

SPL left -3 -50 59 7 6,359 < 0.0001 591

SPL right 2 -64 40 7 6,456 < 0.0001 905

SPL left -6 -61 31 7 6,255 < 0.0001 1088

PreCUN right 6 -48 38 31 5,407 < 0.0001 233

PreCUN right 31 -71 34 31 9,461 < 0.0001 463

CUN left -15 -72 20 30 -6,437 < 0.0001 171

CUN right 12 -72 21 30 -8,095 < 0.0001 316

PCC left -6 -48 23 30 7,308 < 0.0001 1265

PCC right 7 -53 7 30 4,737 < 0.0001 316

PCC left -1 -28 32 23 8,300 < 0.0001 333

PCC left -2 -16 32 23 7,841 < 0.0001 340

MTG right 62 -21 -12 21 6,283 < 0.0001 337

**D 1: Cognitive art evaluation group: right PCC/preCUN at pre-intervention (T0)**

VPFC left -1 51 11 10 19,584 < 0.0001 1884

VPFC right 3 54 13 10 21,539 < 0.0001 1701

IPL left -36 -67 28 39 23,895 < 0.0001 4087

IPL right 44 -59 26 39 27,814 < 0.0001 9172

PCC left -4 -55 29 31 36,410 < 0.0001 15895

PCC right 6 -55 27 31 40,847 < 0.0001 11933

CUN right 19 -78 28 30 16,472 < 0.0001 281

PreCUN left -16 -78 38 7 12,887 < 0.0001 124

MTG right 60 -21 -5 21 18,493 < 0.0001 281

MTG left -58 -8 -10 21 15,084 < 0.0001 119

MTG right 56 -2 -14 21 12,712 < 0.0001 368

**D 2: Cognitive art evaluation group: left PCC/preCUN at pre-intervention (T0)**

MPFC left -33 16 48 8 24,259 < 0.0001 1368

MPFC left -21 36 45 8 22,545 < 0.0001 780

MPFC/DLPFC left -3 45 30 9 17,563 < 0.0001 360

MPFC/DLPFC right 4 55 28 9 20,006 < 0.0001 715

MPFC/DLPFC left -1 54 24 9 18,364 < 0.0001 479

VPFC left -5 47 9 10 18,464 < 0.0001 383

VPFC right 3 54 2 10 21,251 < 0.0001 1005

VPFC left -2 53 3 10 21,200 < 0.0001 1609

IPL left -35 -66 29 39 28,912 < 0.0001 7430

PCC right 5 -55 28 31 32,913 < 0.0001 10152

PCC left -5 -56 28 31 40,995 < 0.0001 14366

IPL right 43 -61 27 39 25,817 < 0.0001 8452

CUN left -5 -90 4 30 14,544 < 0.0001 392

CUN right 17 -80 29 30 19,197 < 0.0001 752

CUN right 6 -86 13 30 19,009 < 0.0001 1524

MTG left -62 -47 0 21 14,845 < 0.0001 121

MTG right 59 -23 -6 21 15,864 < 0.0001 111

MTG left -60 -7 -12 21 15,800 < 0.0001 217

**E 1: Cognitive art evaluation group: right PCC/preCUN at post-intervention (T1)**

PMC right 19 24 51 6 15,968 < 0.0001 113

MPFC/DLPFC left -4 46 28 9 17,767 < 0.0001 480

MPFC/DLPFC right 1 48 25 9 20,469 < 0.0001 820

VPFC left -3 59 4 10 13,217 < 0.0001 94

VPFC right 2 56 12 10 19,493 < 0.0001 732

IPL left -47 -70 11 39 17,395 < 0.0001 570

IPL right 43 -59 27 39 30,285 < 0.0001 7273

PCC left -5 -57 30 31 33,245 < 0.0001 12600

PCC right 6 -55 29 31 47,000 < 0.0001 9446

PCC left -15 -58 14 30 18,900 < 0.0001 300

PreCUN left -29 -72 38 31 15,484 < 0.0001 326

MTG right 59 -32 1 21 15,769 < 0.0001 340

SOG left -35 -74 27 19 17,619 < 0.0001 391

**E 2: Cognitive art evaluation group left PCC/preCUN at post-intervention (T1)**

MPFC left -19 37 45 8 15,100 < 0.0001 146

MPFC/DLPFC right 1 50 19 9 17,658 < 0.0001 239

MPFC/DLPFC left -3 46 23 9 19,269 < 0.0001 496

VPFC right 1 54 -2 10 13,916 < 0.0001 322

**Region Side X Y Z BA t-score *P*-value (corr.) size (mm³)**

VPFC left -2 56 -1 10 13,825 < 0.0001 164

IPL right 42 -64 28 39 23,099 < 0.0001 2082

IPL left -45 -55 29 39 16,737 < 0.0001 359

PreCUN left -31 -72 34 31 21,679 < 0.0001 1161

PCC right 3 -56 30 31 35,285 < 0.0001 8257

PCC left -5 -55 30 31 46,349 < 0.0001 9989

**F 1: Contrast T0 vs.T1: right PCC/preCUN**

SPL left -2 -74 31 7 8,449 < 0.0001 189

PCC left -3 -27 44 31 7,348 < 0.0001 124

**F 2: Contrast T0 vs. T1: left PCC/preCUN**

PMC left -37 14 46 6 -9,391 < 0.0001 262

PMC right 6 34 53 6 -8,841 < 0.0001 153

PMC left -28 17 50 6 -8,604 < 0.0001 495

PMC left -19 27 54 6 -6,482 < 0.0001 130

MPFC right 32 40 38 8 -9,184 < 0.0001 537

MPFC left -17 44 41 8 -9,569 < 0.0001 210

MPFC right 41 13 43 8 -12,735 < 0.0001 766

MPFC right 31 32 44 8 -8,507 < 0.0001 261

MPFC/DLPFC left -32 48 32 9 -6,754 < 0.0001 136

MPFC/DLPFC right 48 22 32 9 -10,425 < 0.0001 730

MPFC/DLPFC left -40 29 37 9 -10,026 < 0.0001 466

MPFC/DLPFC right 5 55 30 9 -7,983 < 0.0001 549

MPFC/DLPFC left -8 57 27 9 -8,584 < 0.0001 334

VPFC right 28 50 27 10 -12,344 < 0.0001 1501

VPFC left -28 58 13 10 -10,391 < 0.0001 503

VPFC right 20 62 22 10 -7,160 < 0.0001 528

VPFC left -26 59 23 10 -9,020 < 0.0001 272

VPFC right 40 56 11 10 -9,694 < 0.0001 622

VPFC left -2 61 9 10 -7,473 < 0.0001 250

VPFC right 4 51 1 10 -9,550 < 0.0001 1159

VLPFC right 50 19 19 45 -9,846 < 0.0001 257

VLPFC left -47 21 2 45 -7,762 < 0.0001 150

DLPFC left -46 46 2 46 -10,954 < 0.0001 635

M1 right 48 -13 45 4 -10,209 < 0.0001 203

M1 left -39 -17 53 4 -8,440 < 0.0001 158

IPL right 49 -38 48 40 -10,006 < 0.0001 197

IPL right 53 -37 36 40 -10,036 < 0.0001 557

IPL left -33 -67 28 39 -10,132 < 0.0001 4951

IPL right 40 -61 26 39 -11,420 < 0.0001 6241

SPL right 5 -32 51 5 -10.681 < 0.0001 340

SPL left -20 -54 55 7 -9,395 < 0.0001 139

SPL right 9 -63 47 7 -9,034 < 0.0001 256

CUN right 9 -90 10 30 -8,828 < 0.0001 548

CUN right 21 -81 20 30 -8,191 < 0.0001 226

CUN right 17 -67 10 30 -8,773 < 0.0001 389

PCC left -3 -57 20 31 -10,540 < 0.0001 6024

PCC right 4 -55 25 31 -8,852 < 0.0001 5444

**Abbreviations**: S1, primary sensosenory cortex; ACC, anterior cingulate cortex; MPFC, medial prefrontal cortex; VPFC, ventral prefrontal cortex; DLPFC, dorsolateral prefrontal cortex; VLPFC, ventrolateral prefrontal cortex, PCC, posterior cingulate cortex; M1, primary motor cortex; PMC; premotor cortex; IPL, inferior parietal lobule; SPL, superior parietal lobule; PreCUN, precuneus; CUN, cuneus; MTG, middle temporal gyrus; STG, superior temporal gyrus; SOG, superior occipital gyrus; RSCC, retrosplenial cingulate cortex.
