# Supplementary material for: How Art Changes Your Brain: Differential Effects of Visual Art Production and Cognitive Art Evaluation on Functional Brain Connectivity
Source: PLoS One. 2014 Jul 1;9(7):e101035. doi: 10.1371/journal.pone.0101035 (PMC4077746; doi:10.1371/journal.pone.0101035)
Supplement: Table S3 — Regions of functional connectivity at rest depicted in Fig. 3 . (DOC) [file pone.0101035.s003.doc]

**Supplementary Table 3**

**Table S3: Correlation between functional connectivity and resilience depicted in Fig. 4**

**Region Side X Y Z BA r *P*-value size (mm³)**

**A: Correlating right PCC/preCUN with resilience at T1**

PMC left -6 10 62 6 0.57 < 0.05 147

PMC right 5 15 60 6 0.60 < 0.05 120

PMC left -35 12 53 6 0.59 < 0.05 304

PMC right 8 33 51 6 0.60 < 0.05 599

MPFC left -31 26 46 8 0.61 < 0.05 176

MPFC left -14 32 49 8 0.62 < 0.05 643

MPFC left -7 45 45 8 0.59 < 0.05 262

MPFC/DLPFC right 51 16 24 9 0.60 < 0.05 465

MPFC/DLPFC right 6 51 31 9 0.60 < 0.05 1019

MPFC/DLPFC left -4 54 26 9 0.58 < 0.05 518

MPFC/DLPFC right 24 40 35 9 0.60 < 0.05 130

VPFC right 30 53 6 10 0.58 < 0.05 235

VPFC left -11 60 21 10 0.60 < 0.05 110

VLPFC left -52 21 18 45 0.60 < 0.05 615

VLPFC right 43 28 -2 47 0.60 < 0.05 349

VLPFC left -47 35 0 45 0.58 < 0.05 547

ACC left -1 48 8 32 0.58 < 0.05 262

STG left -49 -53 21 22 0.63 < 0.05 296

STG right 50 -53 19 22 0.59 < 0.05 182

MTG right 56 -32 -3 21 0.59 < 0.05 114

IPL left -46 -38 39 40 -0.58 < 0.05 177

IPL right 59 -24 32 40 -0.58 < 0.05 114

IPL left -39 -47 41 40 -0.60 < 0.05 144

PCC right 14 -37 23 31 -0.58 < 0.05 323

PCC left -8 -29 22 23 -0.57 < 0.05 116

INS right 36 -5 22 13 -0.60 < 0.05 197

**A: Correlating left PCC/preCUN with resilience at T1**

PMC right 20 -1 58 6 0.60 < 0.05 180

MPFC left -17 33 42 8 0.59 < 0.05 247

MPFC right 6 48 41 8 0.58 < 0.05 369

MPFC left -10 36 50 8 0.61 < 0.05 146

MPFC right 5 34 51 8 0.58 < 0.05 221

MPFC/DLPFC right 24 41 36 9 0.58 < 0.05 189

MPFC/DLPFC left -6 56 27 9 0.58 < 0.05 408

MPFC/DLPFC right 13 57 27 9 0.63 < 0.05 150

MPFC/DLPFC right 5 50 31 9 0.59 < 0.05 588

VLPFC right 50 19 23 45 0.59 < 0.05 327

VLPFC left -51 24 15 45 0.59 < 0.05 185

PCC right 4 -60 9 30 0.61 < 0.05 115

STG right 49 -52 17 22 0.61 < 0.05 412

STG left -49 -53 23 22 0.60 < 0.05 258

STG right 62 -17 0 22 0.61 < 0.05 136

MTG right 58 -29 0 21 0.59 < 0.05 272

SOG right 34 -75 24 19 0.60 < 0.05 640

**Abbreviations**: S1, primary sensosenory Cortex; ACC, anterior cingulate cortex; MPFC, medial prefrontal cortex; VPFC, ventral prefrontal cortex; DLPFC, dorsolateral prefrontal cortex; VLPFC, ventrolateral prefrontal cortex, PCC, posterior cingulate cortex; PMC, premotor cortex; IPL, inferior parietal lobule; MTG, middle temporal gyrus; STG, superior temporal gyrus; SOG, superior occipital gyrus.
