# Supplementary material for: How Art Changes Your Brain: Differential Effects of Visual Art Production and Cognitive Art Evaluation on Functional Brain Connectivity
Source: PLoS One. 2014 Jul 1;9(7):e101035. doi: 10.1371/journal.pone.0101035 (PMC4077746; doi:10.1371/journal.pone.0101035)
Supplement: Text S1 — Estimation of missing data with an analysis of regression. (DOC) [file pone.0101035.s005.doc]

**Text S1. Estimation of missing data with an analysis of regression.**

**Statistical analysis**. Overall, three values of participants in the visual art production group were missing (two participants of T1 and one participants of T0). Missing data were estimated with an analysis of regression that relied on external predictors of personality constructs (i.e., Big Five, sense of meaningfulness (sense of coherence; SOC)) [1].

For the replacement of T0 the following regression-model was used: Resilience value T0 = 10.569 + (1.440 * T0 meaningfulness, SOC) + (0.768 * T0 extraversion, Big Five Inventory, German Socio-Economic Panel (SOEP)) + (0.745 * T0 openness, Big Five Inventory, SOEP).

For the replacement of T1 the following regression-model was used: Resilience value T1 = 14.650 + (1.498 * T1 meaningfulness, SOC) + (0.574 * T1 extraversion, Big Five Inventory SOEP) + (0.653 * T1 openness, Big Five Inventory SOEP).

**REFERENCES**

1. Cohen J, Cohen P (1975) Applied Multiple Regression/Correlation Analysis for the Behavioral Sciences. Hillsdale, NJ: Erlbaum.
