# Supplementary material for: Correction: How Art Changes Your Brain: Differential Effects of Visual Art Production and Cognitive Art Evaluation on Functional Brain Connectivity
Source: PLoS One. 2014 Dec 22;9(12):e116548. doi: 10.1371/journal.pone.0116548 (PMC4274162; doi:10.1371/journal.pone.0116548)
Supplement: Table S2 — Regions of functional connectivity depicted in Fig. 2. (DOC) [file pone.0116548.s001.doc]

**Table S2. Regions of functional connectivity depicted in Fig. 2**

**Region Side X Y Z BA t-score *P*-value (corr.) size (mm³)**

**A: Visual art production group: left VisCX at T0**

VisCx left -7 -72 14 18 42,068 < 0.0001 12315

VisCx right 11 -72 16 18 37,811 < 0.0001 17133

preCUN left -26 -65 39 7 20,093 < 0.0001 389

preCUN left -19 -70 41 7 16,870 < 0.0001 149

STG right 48 -58 16 22 23,940 < 0.0001 1453

STG left -59 -48 14 22 15,620 < 0.0001 117

MTG left -44 -64 10 37 27,623 < 0.0001 2181

MTG left -62 -39 3 22 14,482 < 0.0001 654

TTG left -50 -23 11 42 16,789 < 0.0001 161

**B: Visual art production group: right VisCX at T0**

VisCx left -6 -72 12 18 37,872 < 0.0001 5237

VisCx right 7 -73 13 18 49,974 < 0.0001 8715

MOG right 42 -67 8 19 29,205 < 0.0001 1505

MOG right 27 -90 6 18 15,898 < 0.0001 111

INS right 58 -36 15 13 22,228 < 0.0001 520

PCC right 18 -72 28 31 30,922 < 0.0001 1309

CUN left -22 -78 14 17 20,158 < 0.0001 353

STG left -59 -44 8 22 19,671 < 0.0001 423

MTG left -42 -67 16 21 24,603 < 0.0001 1023

MTG left -48 -57 8 21 20,322 < 0.0001 363

TTG right 55 -14 8 42 21,594 < 0.0001 340

**A: Cognitive art evaluation group: left VisCX at T0**

VisCx left -10 -76 15 18 39,605 < 0.0001 22781

VisCx right 10 -76 15 18 35,827 < 0.0001 30896

MOG left -44 -75 6 19 24,317 < 0.0001 1860

precUN right 2 -56 36 7 19,979 < 0.0001 2989

preCUN left -1 -53 46 7 17,450 < 0.0001 1137

TTG right 62 -15 11 42 18,979 < 0.0001 308

STG left -56 -23 11 41 21,083 < 0.0001 296

STG right 54 -13 6 22 16,169 < 0.0001 207

STG left -57 -12 6 22 18,594 < 0.0001 338

MTG right 47 -58 10 21 23,053 < 0.0001 1966

**B: Cognitive art evaluation group: right VisCX at T0**

VisCx left -9 -72 10 18 43,515 < 0.0001 19604

VisCx right 10 -73 9 18 46,660 < 0.0001 21493

MOG left -47 -70 6 19 19,733 < 0.0001 867

MOG left -37 -83 5 19 19,507 < 0.0001 422

preCUN right 27 -64 36 7 19,507 < 0.0001 1123

preCUN left -37 -58 41 7 14,186 < 0.0001 114

preCUN right 2 -54 46 7 18,553 < 0.0001 1767

preCUN left -2 -52 50 7 17,824 < 0.0001 1215

CUN right 15 -75 26 18 28,930 < 0.0001 10677

CUN left -15 -77 24 18 19,986 < 0.0001 8810

MTG right 46 -58 10 21 23,697 < 0.0001 2501

MTG left -43 -62 13 21 20,897 < 0.0001 477

STG right 59 0 3 22 15,715 < 0.0001 301

**Abbreviations**: VisCx, visual cortex; PCC, posterior cingulate cortex; INS, insula; MTG, middle temporal gyrus; STG, superior temporal gyrus; MOG, middle occipital gyrus; SOG, superior occipital gyrus; TTG, tranversal temporal gyrus.
