# Supplementary material for: Correction: How Art Changes Your Brain: Differential Effects of Visual Art Production and Cognitive Art Evaluation on Functional Brain Connectivity
Source: PLoS One. 2014 Dec 22;9(12):e116548. doi: 10.1371/journal.pone.0116548 (PMC4274162; doi:10.1371/journal.pone.0116548)
Supplement: Table S3 — Regions of functional connectivity at rest depicted in Fig. 3. (DOC) [file pone.0116548.s002.doc]

**Table S3. Regions of functional connectivity at rest depicted in Fig. 3**

**Region Side X Y Z BA t-score *P*-value (corr.) size (mm³)**

**A: Visual art production group: left S1/M1 at T0**

S1 left -36 -29 46 3 37,306 < 0.0001 16655

S1 right 57 -17 22 3 11,941 < 0.0001 198

PMC left -30 -5 49 6 17,611 < 0.0001 272

PMC right 39 -10 51 6 15,825 < 0.0001 169

PMC left -51 -6 31 6 25,604 < 0.0001 2047

PMC right 3 -11 53 6 24,274 < 0.0001 2422

PMC left -1 -12 53 6 25,537 < 0.0001 4731

MPFC/DLPFC right 57 3 21 9 13,343 < 0.0001 123

M1 right 25 -24 61 4 18,687 < 0.0001 597

IPL right 40 -27 45 40 29,442 < 0.0001 6601

SPL left 1 -48 49 7 22,489 < 0.0001 3502

SPL right 21 -52 49 7 23,846 < 0.0001 3051

SPL left -26 -56 46 7 17,222 < 0.0001 230

SPL right 15 -74 31 7 13,251 < 0.0001 155

MTG left -46 -59 -4 19 17,141 < 0.0001 519

**B: Visual art production group: left S1/M1 at T1**

S1 left -35 -32 46 3 37,576 < 0.0001 2898

M1 right 32 -21 59 4 16,884 < 0.0001 543

M1 left -56 -13 35 4 17,987 < 0.0001 267

M1 right 54 -13 39 4 17,404 < 0.0001 286

IPL right 30 -40 56 40 20,491 < 0.0001 500

SPL right 24 -48 56 7 14,209 < 0.0001 121

SPL left -3 -40 59 5 16,279 < 0.0001 275

SPL right 3 -38 61 5 15,923 < 0.0001 221

SPL left -3 -54 48 7 15,092 < 0.0001 106

SPL right 18 -67 42 7 17,210 < 0.0001 350

PreCUN left -22 -67 36 7 14,920 < 0.0001 123

PreCUN right 29 -75 20 31 17,126 < 0.0001 595

PMC left -1 -19 61 6 16,001 < 0.0001 340

**C: Contrast T0 vs. T1: left S1/M1**

S1 left -32 -30 46 3 -14,724 < 0.0001 26913

PMC left -21 -1 56 6 -7,212 < 0.0001 2567

PMC right 16 5 61 6 -7,186 < 0.0001 1043

PMC left -50 -5 27 6 -12,343 < 0.0001 7186

PMC right 5 -12 52 6 -10,611 < 0.0001 4458

PMC left -1 -8 51 6 -11,538 < 0.0001 7168

MPFC left -26 28 41 8 -7,667 < 0.0001 1125

MPFC left -10 41 38 8 -8,793 < 0.0001 661

MPFC/DLPFC right 47 5 20 9 -12,643 < 0.0001 4964

MPFC/DLPFC left -30 32 25 9 -7,498 < 0.0001 276

MPFC/DLPFC right 23 48 36 9 -8,192 < 0.0001 799

MPFC/DLPFC right 28 32 38 9 -7,901 < 0.0001 865

VPFC left -34 48 22 10 -6,796 < 0.0001 772

VPFC right 30 56 21 10 -7,302 < 0.0001 399

DLPFC left -36 30 10 46 -9,228 < 0.0001 259

IPL right 35 -31 43 40 -13,254 < 0.0001 20427

IPL left -39 -54 31 39 -10,377 < 0.0001 2707

SPL left -17 -58 39 7 -13,139 < 0.0001 7359

SPL right 10 -60 40 7 -8,727 < 0.0001 6882

SPL left -4 -43 44 7 -12,008 < 0.0001 6061

SPL right 9 -47 47 7 -10,661 < 0.0001 3142

ACC right 5 33 27 32 -5,500 < 0.0001 158

ACC left -7 29 26 32 -4,030 < 0.0001 123

MTG right 56 -34 2 22 -9,216 < 0.0001 1043

Th right 10 -6 15 - -8,930 < 0.0001 776

Th left -13 -10 16 - -7,443 < 0.0001 855

**Region Side X Y Z BA t-score *P*-value (corr.) size (mm³)**

**D: Cognitive art evaluation left S1/M1 seed at pre-intervention (T0)**

S1 right 38 -32 50 3 27,500 < 0.0001 1997

S1 left -51 -17 35 3 31,205 < 0.0001 1969

S1 right 37 -20 47 3 24,556 < 0.0001 537

S1 left -34 -30 47 3 47,383 < 0.0001 5339

PMC right 1 -31 60 6 19,280 < 0.0001 296

PMC left -3 -38 59 6 21,598 < 0.0001 757

PMC right 27 -17 63 6 18,710 < 0.0001 293

PMC left -27 -18 61 6 24,837 < 0.0001 616

PreCUN right 9 -46 57 7 23,697 < 0.0001 269

PreCUN left -20 -54 50 7 28,530 < 0.0001 1661

PreCUN right 22 -54 50 7 23,770 < 0.0001 1375

PreCUN left -16 -78 25 7 25,173 < 0.0001 1805

PreCUN right 23 -74 25 31 24,106 < 0.0001 2763

PreCUN left -28 -63 34 7 15,288 < 0.0001 237

PCC right 15 -66 14 31 17,095 < 0.0001 196

PCC left -28 -70 21 31 15,302 < 0.0001 136

MOG left -27 -79 11 19 15,956 < 0.0001 350

MTG left -49 -67 4 37 17,154 < 0.0001 182

MTG right 47 -63 14 37 16,156 < 0.0001 242

**E: Cognitive art evaluation S1/M1 seed at post-intervention (T1)**

S1 left -35 -31 51 3 38,963 < 0.0001 5668

S1 left -59 -14 23 3 19,010 < 0.0001 436

S1 right 35 -22 45 3 22,092 < 0.0001 319

PMC left -2 -23 60 6 17,681 < 0.0001 622

PCC right 4 -17 44 31 16,600 < 0.0001 170

IPL right 31 -38 53 40 24,471 < 0.0001 4424

CUN left 17 -85 21 18 20,353 < 0.0001 1159

CUN right 19 -83 22 18 13,649 < 0.0001 221

PreCUN left -25 -52 51 7 29,970 < 0.0001 1416

PreCUN right 14 -61 46 7 24,469 < 0.0001 3202

PreCUN left -19 -66 42 7 16,401 < 0.0001 414

PreCUN right 3 -53 53 7 17,441 < 0.0001 248

SPL left -7 -42 59 5 20,929 < 0.0001 670

SPL right 2 -40 59 5 18,175 < 0.0001 759

MOG right 38 -74 7 19 15,442 < 0.0001 165

MOG left -28 -72 37 19 12,461 < 0.0001 289

MTG left -42 -62 9 37 13,704 < 0.0001 117

**F: Contrast left S1/M1 seed T0 vs. T1**

S1 left -28 -31 47 3 -12,289 < 0.0001 2642

S1 right 53 -18 33 2 -10,252 < 0.0001 535

S1 left -42 -22 35 3 -12,070 < 0.0001 3405

S1 right 30 -34 43 3 -5,841 < 0.0001 118

M1 right 19 -24 51 4 -11,563 < 0.0001 199

PMC left -45 -7 37 6 -8,857 < 0.0001 1342

PMC right 29 -13 57 6 -11,307 < 0.0001 1636

PMC left -24 -17 62 6 -10,389 < 0.0001 1608

PMC right 44 1 44 6 -9,036 < 0.0001 207

PMC right 8 -29 65 6 -6,810 < 0.0001 149

MPFC right 28 20 45 8 -7,861 < 0.0001 287

MPFC/DLPFC left -19 42 36 9 -8,201 < 0.0001 172

MPFC/DLPFC right 28 46 32 9 -7,351 < 0.0001 218

VPFC left -17 61 11 10 -10,627 < 0.0001 1037

VPFC left -36 50 9 10 -4,730 < 0.0001 128

VPFC left -4 54 0 10 -5,777 < 0.0001 377

ACC right 4 42 -2 24 -7,876 < 0.0001 574

ACC left -8 -1 37 24 -8,910 < 0.0001 346

ACC right 5 -20 38 24 -11,060 < 0.0001 588

ACC left -11 -20 35 24 -5,931 < 0.0001 138

ACC right 5 11 33 24 -7,040 < 0.0001 206

ACC left -4 24 27 32 -6,463 < 0.0001 124

PCC right 5 -65 16 31 -9,463 < 0.0001 4900

**Region Side X Y Z BA t-score *P*-value (corr.) size (mm³)**

PCC left -17 -70 25 31 -10,204 < 0.0001 5601

PCC right 31 -71 22 31 -8,134 < 0.0001 4280

PreCUN left -5 -69 42 7 -7,517 < 0.0001 350

PreCUN right 2 -59 41 7 -6,986 < 0.0001 758

SPL left -28 -55 45 7 -11,673 < 0.0001 1601

SPL right 20 -54 50 7 -6,768 < 0.0001 566

IPL left -31 -46 54 40 -7,044 < 0.0001 666

IPL right 57 -43 31 40 -8,283 < 0.0001 430

IPL right 36 -34 56 40 -6,231 < 0.0001 202

IPL right 42 -34 42 40 -9,655 < 0.0001 190

IPL right 37 -48 47 40 -9,949 < 0.0001 137

IPL right 29 -55 37 39 -7,356 < 0.0001 160

STG left -55 -9 9 22 -6,887 < 0.0001 277

MOG right 27 -79 14 19 -6,818 < 0.0001 2061

MOG left -27 -78 11 19 -7,347 < 0.0001 1000

MTG right 45 -51 -2 21 -5,431 < 0.0001 235

MTG left -50 -65 0 21 -9,024 < 0.0001 754

**Abbreviations**: S1, primary sensosenory Cortex; M1, primary motor cortex; ACC, anterior cingulate cortex; MPFC, medial prefrontal cortex; VPFC, ventral prefrontal cortex; DLPFC, dorsolateral prefrontal cortex; VLPFC, ventrolateral prefrontal cortex; PMC; premotor cortex; MTG, middle temporal gyrus; MOG, middle occipital gyrus; PreCUN, precuneus; Th, thalamus; IPL, inferior parietal lobule; SPL, superior parietal lobule.
